# Vestiges of the Natural History of Creation

**Published:** 1845-01

**Authors:** 


### Art. XVI.

*Vestiges of the Natural History of Creation.—Lond.* 1844. 8vo, pp. 390.

This is a very beautiful and a very interesting book. Its theme is one of the grandest that can occupy human thought—no less than the creation of the universe. But neither its interest nor its grandeur would of themselves claim our notice, in this Journal, were not the science of Physiology called in to play an important part in the elaboration of the author's theory. Therefore it is, mainly, that we propose to make it the subject of a critical analysis; though we are also influenced by the abstract desire to place before our readers matter for their contemplation, which cannot fail at once to elevate, to gratify and to enrich the mind. It has always been one of the boasts of our noble profession that it touches and blends with every science; and we should be sorry that our humble efforts should at any time be wanting to stimulate its professors to exertions that might still justify the boast.

It will be our duty to show that the " Vestiges" contains many defects and not a few errors ; but it will also be our delight to prove, from its own eloquent pages, that its beauties and its merits are more numerous still.

In the First Chapter,—" *On the Bodies of Space, their Arrangement and Formation,*—a rapid but masterly sketch is given of the Nebular Hypothesis, as developed by Herschel (the elder) and Laplace. As it is probable that many of our readers may be unpossessed of a clear conception of this doctrine, and as an acquaintance with it is essential to the comprehension of our author's subsequent argument, we shall endeavour to convey to them, in as few words as possible, an idea of its nature.

When a powerful telescope is directed to the heavens, not only does it bring into view innumerable multitudes of stars, which were previously invisible, but also many cloudy patches, of varying forms, and of greater or less extent, which are termed *nebulæ.* Of these nebulæ, some may be *resolved,* by telescopes of still higher power, into distinct stars; just as a telescope of very moderate power suffices to *resolve* the milky way, which presents a nebular appearance to the naked eye. There are others, which have been hitherto considered *irresolvable* ; but which, by the aid of telescopes of still higher power, will be most probably resolved like the preceding. These clusters seem to be distributed through space at varying distances; and every one of them may be regarded as a repetition of that firmament of our own, which includes *all the distinct stars* with which the telescope makes us acquainted.—But besides *these* nebulæ, there is a class distinguished by certain peculiarities of appearance, which seem to preclude the notion of their being formed by the aggregation of distinct stars ; and the idea was propounded (we believe) by Sir W. Herschel, that they consist of a diffused self-luminous matter. In some of these, small bright *nuclei* present themselves, studding their surface here and there ; as if the diffused matter was becoming condensed in certain points. And in others, this condensation has proceeded so far, as almost to break up the entire mass into divisions, every one containing a nucleus. These seem to be stages of transition towards the production of distinct stars.

Again, it was observed by Sir W. Herschel, that among the fixed stars of our own firmament, there are considerable variations of appearance : some of them being definite points of clear and brilliant light ; others being surrounded with a slight luminosity ; others, again, having a more extended luminous atmosphere; in others the central nucleus being scarcely visible; and others presenting no other appearance than that of a circular disc, of which the light, faint at the edges, *gradually* increases in intensity towards the centre. Hence it was supposed by Sir W. Herschel, that all the bodies composing the stellar universe have been formed by the gradual condensation of *nebular matter ;* and that the appearances just described indicate so many stages in this condensation.

How far La Place was indebted to Herschel for this idea, we do not know ; but, starting from the same fundamental conception of a diffused *nebular matter* as the original condition of the universe, he set himself to calculate, by the aid of mathematical formulæ, in what manner the condensation of this matter, by the mutual attraction of its particles, would operate in producing,—not merely suns or stars,—but *systems* of planets revolving round the suns, and satellites around these primaries. It can easily be shown by reference to well-known physical phenomena, as well as by mathematical investigation, that the *rush* of the nebular matter towards a centre, would produce a rotatory motion in the mass,—

that the rapidity of this motion would increase, as, by the condensation of the mass, its bulk diminishes,—that, in this process of condensation, the outer part would be very probably thrown off in the form of a ring, —that this ring, continuing to revolve at the rate which the whole mass possessed at the period of its detachment, *might possibly* remain as such, or *might* break into several portions, but would *most probably* become itself condensed into one mass, by the attraction of its whole material towards the thickest part of it, thus forming a Planet,—that this process would probably be repeated several times, thus forming a series of planets, and that, at last, the process of condensation would end in the formation of a centre or Sun, revolving on its axis at a rate more rapid than that of the orbital revolutions of any of its attendant planets, and of a series of planets revolving around it in times bearing a certain definite proportion to their distances. Further, in the condensation of the detached ring into a globular mass, the rush of nebular matter towards the point of greatest density would impress on *it* also a rotatory motion, whilst its orbital revolution around the first centre continued; and in the process of *its* condensation, rings would be detached, which also would probably condense into globular masses, having a revolution round it and a rotation on their own axis, as in the preceding case,— thus forming the Satellites of the primary planets.

All this, it will be seen, has such a perfect correspondence with the actual condition of our solar system, that it has the appearance of being an hypothesis constructed with express reference to its phenomena; but so far is this from being the case, that these phenomena might be foretold with almost the same accuracy as *any* Astronomical prediction, in regard to any other mass of nebular matter in process of condensation; and we may fully calculate upon finding the very same laws holding good, in regard to the proportions between the times and the distances of revolution, in *any other* planetary system,—should the improvement in our means of observation ever allow such to be discovered,—as in our own. Even the apparent exceptions,—those furnished by double and triple stars for example, which are supposed to result from the consolidation of the first or primary nebular mass around two or three distinct centres, instead of one, the bodies thus formed having a revolution around their common centre of gravity,—that presented in the consolidation of the ring thrown off between Mars and Jupiter, into four small planets,—and that afforded by the persistence of the annular form, in the ring detached from the body of Saturn, during its condensation, subsequently to the production of its nearest satellite,—add strength to the case; since they are not only accounted for by it, but are shown to be *probable* occurrences.— Further, there is evidence that our sun, if seen from afar, would present the appearance of a slightly-nebulous star; the phenomenon termed the zodiacal light, appearing to indicate, that he is surrounded by a lenticular band of self-luminous matter of extreme tenuity, extending beyond the orbit of Venus.

A very important confirmation of this hypothesis has been recently afforded by the calculations of M. Comte; who has set himself to determine whether, supposing that the mass which is now condensed in the Sun could be again expanded as far as the orbit of Mercury, its time of rotation would be the same with that, in which Mercury now executes his orbital revolution ; and to solve the same problem in regard to the times of revolution of all the other planets round the sun, and of the secondaries round their primaries. The coincidence between the *calculated* and the *observed* time is so close in the case of the moon, that the two agree within an hour or two. The difference is somewhat greater in the case of the planets, but still does not exceed a forty-fifth part of the amount, and it may be fairly imputed to the neglect or inaccuracy of some small data involved in the calculation.

This is an outline view of the celebrated Nebular hypothesis; which, we believe, the greatest minds of our time feel disposed to regard as the expression of the *probable* history of the production of the universe as we now behold it. Our author, perhaps, expresses himself a little more strongly on the subject than *we* feel justified in doing, when he says that " the nebular hypothesis is supported by so many ascertained features of the celestial scenery, and by so many calculations of exact science, that it is impossible for a candid mind to refrain from giving it a cordial reception, if not to repose full reliance upon it, even without seeking for it support of any other kind." (p. 20.) If it were *certain* that the data which have been assumed, as the results of observation, are mainly correct, we should be quite ready to receive the hypothesis with the cordiality which he claims for it; but at a time when the scientific world is awaiting, with anxious expectation, the revelations of a new instrument of surpassing perfection (Lord Rosse's telescope), we deem it unwise to assume that there is a single clearly-established example of the existence of uncondensed nebular matter; every improvement in the construction of the telescope having led to the *resolution* of nebulæ, which were previously deemed irresolvable. And it is, we presume, on this account, that those who are most entitled to give an opinion upon the subject, express themselves with caution, and decline to receive the doctrine in any other way than as a *probable* hypothesis. But even if *this* cosmogony should be overturned by the results of newer observations, we cannot doubt that another of equal simplicity and comprehensiveness will be substituted for it; and in the mean time it may be entertained as the nearest approach yet made by the human mind to the conception of the mode, in which the physical universe has been called into being.

But does this explanation in any way preclude the idea of the operation of Creative power, in the formation of these countless myriads of suns and systems, of which astronomy takes cognizance? It is said to have been framed by La Place, with the express purpose of " doing away with the necessity for a Creator," in conformity with the strangely unphilosophical spirit prevalent in his time, which considered the belief in a Deity as an antiquated fiction, adapted only to the credulity of the ignorant, and the knavery of the interested. We cannot but deeply lament, that the author of the ' Nebular Hypothesis,' and the ' Demonstrator of the Stability of the Solar System,' should have been so blind to the real tendency of his views; and that, after having ascended in sublime generalization so near to the First Cause, he should have made no attempt to ascend still higher, by inquiring into the origin of nebular matter itself, the source of its properties, and the conditions under which these came into action. So far from admitting the atheistical tendency which timid religionists have attributed to the ' Nebular Hypothesis,' we consider it the grandest contribution which Science has yet made to Religion; by giving us a clearer and more definite comprehension, than any other point of view affords to us, of the omnipotence and omniscience of the Creator. We *feel* that the mind, which could at a glance foresee the whole operation of the mutual attraction of the particles of nebular matter, as it presents itself in our system alone, and which could provide, as the necessary result of that same principle, the wonderful system of checks and counterchecks that ensures its stability, must have a capacity far beyond caparison with our own; and when it is considered that the wide sweep of Uranus, nearly twelve thousand millions of miles in length, includes what is but a point in the vast universe of which we take cognizance, and that in every part the same phenomena are probably going on, we *feel* that the mind which devised all this can be nothing less than Infinite.

Taking the truth of the 'Nebular Hypothesis' for granted, our author founds upon it two inferences, which constitute the basis of his subsequent argument. The first of these has reference to the simple question of the *history* of the universe; the second to the *cause* of the sequence of phenomena which it embraces. "The first idea," he remarks (p. 21,) "which all this impresses upon us is, that the formation of the bodies in space is *still, and at present, in progress*." We shall presently see his application of this idea, and shall not now dwell upon it. We shall give his second deduction in his own clear and striking language.

"Another and more important consideration arises from the hypothesis, namely, as to the means by which the grand process is conducted. The nebulous matter collects around nuclei by virtue of the law of attraction. The agglomeration brings into operation another physical law, by force of which the separate masses of matter are either made to rotate singly, or, in addition to that single motion, are set into a coupled revolution in ellipses. Next centrifugal force comes into play, flinging off portions of the rotating masses, which become spheres by virtue of the same law of attraction, and are held in orbits of revolution round the central body by means of a composition between the centrifugal and gravitating forces. All we see is done by certain laws of matter, so that it becomes a question of extreme interest, what are such laws? All that can yet be said in answer, is, that we see certain natural events proceeding in an invariable order under certain conditions, and thence infer the existence of some fundamental arrangement, which, for the bringing about of these events, has a force and certainty of action similar to, but more precise and unerring than, those arrangements which human society makes for its own benefit, and calls laws. It is remarkable of physical laws, that we see them operating on every kind of scale as to magnitude, with the same regularity and perseverance. The tear that falls from childhood's cheek is globular through the efficacy of that same law of mutual attraction of particles which made the sun and planets round. The rapidity of Mercury is quicker than that of Saturn, for the same reason that, when we wheel a ball round by a string, and make the string wind up round our fingers, the ball always flies quicker and quicker as the string is shortened. Two eddies in a stream, as has been stated, fall into a mutual revolution at the distance of a couple of inches, through the same cause which makes a pair of suns link in mutual revolution at the distance of millions of miles. There is, we might say, a sublime simplicity in this indifference of the grand regulations to the vastness or minuteness of the field of their operation. Their being uniform, too, throughout space, as far as we can scan it, and their being so unfailing in their tendency to operate, so that only the proper conditions are presented, afford to our minds matter for the gravest consideration. Nor should it escape our careful notice, that the regulations on which all the laws of matter operate, are established on a rigidly accurate mathematical basis. Proportions of numbers and geometrical figures rest at the bottom of the whole. All these considerations, when the mind is thoroughly prepared for them, tend to raise our ideas with respect to the character of physical laws, even though we do not go a single step further in the investigation. But it is impossible for an intelligent mind to stop there. We advance from law to the cause of law, and ask, What is that? Whence have come all these beautiful regulations? Here science leaves us, but only to conclude, from other grounds, that there is a First Cause, to which all others are secondary and ministrative, a primitive almighty will, of which these laws are merely the mandates. That great Being, who shall say where is his dwelling place, or what his history! Man pauses breathless at the contemplation of a subject so much above his finite faculties, and only can wonder and adore!" (pp. 23-6.)

We can scarcely imagine that any sane mind, seriously attentive to the question, can take any other view of the character of the Laws of Nature, than that which is so well set forth in the foregoing extract. They are but the expressions of the will of that Divine Being, whose immutability is nowhere more clearly manifested, than in that continuance of the same mode of action,—not merely through the limited period of human experience, but, as we can now comprehend, from the very commencement of the present system of the universe,—which enables us to discern somewhat of the plan on which he acted. If every action of the Creator were to be *immediately* prompted by *present* contingencies, instead of being the result of predetermination based on perfect knowledge of the future, there could be no *law*. If that knowledge of the future were, like man's, imperfect,—though we might trace a general principle when the arrangements were viewed in their totality, the *details* would have much of that unsteadiness and occasional want of consistency, which we perceive in the actions of even the best-regulated human mind. The *laws* would be made to bend to the necessities of the time; and new interpositions would be continually necessary to correct the errors which would arise in the working of the machine. So far, however, is this from being the case, that, in the only instance in which we have been able, from the simplicity of the phenomena, to attain to a complete generalization of them, we have every reason to believe that *the same laws have been in operation from the beginning*, or, in other words, that the work of creation was commenced upon a plan so perfect, as to need no subsequent change.

But whilst we deprecate the idea of constant *interference*, we must not lose sight of the continual *sustaining action* of the Deity. The tendency of some of those philosophers who have most profoundly investigated the phenomena of Nature, has been to imagine that the creation of matter, endowed with certain properties, and therefore subject to certain actions, was the *final* act of the Deity, instead of the *mere commencement* of his operations, as far as the present system of things is concerned. We need not point out how inconsistent this doctrine is with all that Revelation teaches us of the Creator's dealings; but we shall simply say in regard to it, that it appears to us equally opposed to the dictates of sound philosophy. For if it be admitted that matter owes its origin and properties to the Deity, in other words, that its *first* existence is but an expression of the Divine will, what is its *continued* existence but a continued expression of that same will? The idea to which we are objecting may appear to derive support from a fancied analogy between Divine and human laws; it being supposed that the Divine laws, like the human, having been once enacted, are left to work out their results, not only without the interference, but even without any further concern on the part of the Supreme Power. But so far is even this imperfect analogy from supporting such a view, that it runs in direct opposition to it. For what is a human law, but an expression of the will of the governing power; and what is its action upon the community, but the constant though silent operation of that will? If a sudden convulsion overturn the governing power, the laws cease to exist as controlling agencies; all is anarchy and confusion. If the law is inadequate to produce the desired effects, the human legislator modifies or changes it; but with the Divine legislator, no modification has ever been needed; all the future results of the plan having been so completely foreseen in the first instance, that the plan could be arranged to meet them, just as if they were all present.

Let us sum up with the remark, that the term Law of Nature cannot be understood, either logically or philosophically, as anything else than a *human expression* of the plan or principle, which seems to have been acted on by the Creator in the production of the phenomena with which our limited experience brings us acquainted. *We* have no right, limited alike in our powers of observation, and in the use of our reasoning faculties, to assume that the laws which *we* deduce from our experience are those by which the Deity has thought proper to restrict his operations.

We know not where a more beautiful illustration can be drawn from a scientific operation, in aid of the comprehension of a grand religious truth, than that which Mr. Babbage has adduced in his (so-called) 'Ninth Bridgewater Treatise,' with reference to this subject, and to the question of miracles which grows out of it. This illustration has been quoted by our author in a later part of his work; but we shall introduce it here, as more apposite to the *general* question we are discussing,—the real character of natural laws. We presume that most of our readers are acquainted with the *name* at least of Mr. Babbage's calculating machine; an engine which is put in action by ordinary mechanical means, but which is so constructed as to bring up, by the turning of a wheel, any series of numbers that can be arranged on a definite plan, such as those required for mathematical tables, &c. An observer, watching the numbers as they are presented, may be able to discover the plan on which they are arranged, or, in other words, the *law* on which the machine is acting. Now by a particular adjustment of the machine, it may be made to bring up the consecutive numbers, 1, 2, 3, 4, 5, &c. up to *one hundred millions* (or any other amount); and it is pertinently asked by Mr. Babbage, whether any one who has observed a hundred millions of consecutive phenomena, all following the same law, could avoid the belief, that the operation will continue on the same plan. True to this vast induction, the succeeding term will be one hundred million and one; but the next number, instead of being one hundred million and two, is one hundred million *ten thousand* and two. The next term is larger than we should have anticipated, by *thirty* thousand; the next by *sixty* thousand; the next by *a hundred* thousand; the next by *a thousand thousand;* and so on. Here we might say that a *new law* has come into operation; and a little observation might show us the nature of this law, which is, that the *differences between the tens of thousands* of each two consecutive terms, increase in the same regular proportion as the units of the preceding numbers; for the tens of thousands being represented by 1, 3, 6, 10, 15, 21, 28, &c. their differences are 2, 3, 4, 5, 6, 7, &c. If we continue to observe the numbers presented by the machine, we shall find that for a hundred or even a thousand terms, they continue to follow this new law; but after watching them for 2761 terms, we find that the law fails in the case of the 2762d term. If we continue to observe, we shall then discover another law coming into operation, also dependent upon a similar sequence of numbers, but in a different manner. This will continue through about 1450 terms; when a new law is again introduced, which extends over about 950 terms; and this, too, like all its predecessors, fails, and gives place to other laws, which appear at different intervals,—all resulting from the *first adjustment* of the machine. "Now it must be observed," says Mr. Babbage, "that *the law—that each number presented by the engine is greater by unity than the preceding number*, which law the observer has deduced from an induction of a hundred million instances, *was not the true law that regulated its action;* and that the occurrence of 100,010,002 at the 100,000,002d term was *as necessary a consequence of the original adjustment, and might have been as fully foreknown at the commencement, as was the regular succession of any one of the intermediate numbers to its immediate antecedent.* The same remark applies to the next apparent deviation from the new law, which was founded on an induction of 2761 terms, and also to the succeeding laws, with this limitation only,—that, whilst their consecutive introduction at various definite intervals is a necessary consequence of the mechanical structure of the engine, our knowledge of analysis does not enable us to predict the periods themselves, at which the more distant laws will be introduced." How humbling to the pride of man's reason to find, that even the work of his own hands can execute operations, which his boasted intellect cannot fathom!

We trust that we have now dwelt sufficiently on this topic, to develope the view which we have ourselves long entertained, and which is the fundamental idea of the work before us;—that we cannot really attribute *infinite* knowledge and *infinite* power to the Creator, unless we believe that in the plan of creation which He first laid down, *every* contingency was foreseen and provided for; so that the continued operation of the *same* plan has been all that has been required for the perfect working of the machine; the very existence, as well as the actions, of which are dependent upon His sustaining power, *but all* the operations of which, however changeful and uncertain to our limited apprehension, are to His all-seeing glance uniform and definite. And as a corollary to this proposition we would add, that we must not place too much confidence in any law deduced from the results of our own limited experience; when this appears to run counter to the general plan of the Divine operations.

Returning now to our author, we shall briefly follow him through the succeeding chapters, in which he prepares the way for the development of his hypothesis respecting the creation of organized beings. He first takes a brief survey of the "constituent materials of the earth, and of the other bodies of space;" in which he argues from the Nebular hypothesis, that these must be of the same kind throughout, although they may differ in combination and in mode of aggregation; and surmises that, as each planet is passing, like the earth, through a series of changes, dependent upon the gradual cooling of the mass, we have no reason to suppose that it must ever remain, or have remained, destitute of organic life, merely because its present condition seems to us to be inconsistent with the idea. His general views on this subject will be gathered from the two following extracts.

"The earth and all its various substances have at present a certain volume in consequence of the temperature which actually exists. When, then, we find that its matter and that of the associate planets was at one time diffused throughout the whole space, now circumscribed by the orbit of Uranus, we cannot doubt, after what we know of the power of heat, that the nebulous form of matter was attended by the condition of a very high temperature. The nebulous matter of space, previously to the formation of stellar and planetary bodies, must have been a universal fire-mist; an idea which we can scarcely comprehend, though the reasons for arriving at it seem irresistible. The formation of systems out of this matter implies a change of some kind with regard to the condition of the heat. Had this power continued to act with its full original repulsive energy, the process of agglomeration by attraction could not have gone on. We do not not know enough of the laws of heat to enable us to surmise how the necessary change in this respect was brought about, but we can trace some of the steps and consequences of the process. Uranus would be formed at the time when the heat of our system's matter was at the greatest, Saturn at the next, and so on. Now this tallies perfectly with the exceeding diffuseness of the matter of those elder planets, Saturn being not more dense or heavy than the substance cork. It may be that a sufficiency of heat still remains in those planets to make up for their distance from the sun, and the consequent smallness of the heat which they derive from his rays. And it may equally be, since Mercury is twice the density of the earth, that its matter exists under a degree of cold, for which that planet's large enjoyment of the sun's rays is no more than a compensation. Thus there may be, upon the whole, a nearly equal experience of heat amongst all these children of the sun. Where, meanwhile, is the heat once diffused through the system, over and above what remains in the planets? May we not rationally presume it to have gone to constitute that luminous envelope of the sun, in which his warmth-giving power is now held to reside? It could not be destroyed—it cannot be supposed to have gone off into space—it must have simply been reserved to constitute at the last a means of sustaining the many operations of which the planets were destined to be the theatre.

"The tendency of the whole of the preceding considerations is to bring the conviction that our globe is a specimen of all the similarly-placed bodies of space, as respects its constituent matter, and the physical and chemical laws governing it, with only this qualification, that there are *possibly* shades of variation with respect to the component materials, and *undoubtedly* with respect to the conditions under which the laws operate, and, consequently, the effects which they produce. Thus, there may be substances here which are not in some other bodies, and substances here solid may be elsewhere liquid or vaporiform. We are the more entitled to draw such conclusions, seeing that there is nothing at all singular or special in the astronomical situation of the earth. It takes its place third in a series of planets, which series is only one of numberless other systems, forming one group. It is strikingly—if I may use such an expression—a member of a democracy. Hence, we cannot suppose that there is any peculiarity about it which does not probably attach to multitudes of other bodies—in fact, to all that are analogous to it in respect of cosmical arrangements." (pp. 30-3 )

"The characteristics of the moon forbid the idea that it can be at present a theatre of life, like the earth, and almost seem to declare that it never can become so. But we must not rashly draw any such conclusions. The moon may be only in an earlier stage of the progress through which the earth has already gone. The elements which seem wanting may be only in combinations different in those which exist here, and may yet be developed as we here find them. Seas may yet fill the profound hollows of the surface; an atmosphere may spread over the whole. Should these events take place, meteorological phenomena, and all the phenomena of organic life, will commence, and the moon, like the earth, will become a green and inhabited world." (p. 39-40.)

On this last, however, we must take leave to remark, that the laws of heat would seem to indicate that the moon *has already passed* through the present condition of the earth; since smaller bodies cool faster (cæteris paribus) than larger. The author has already adverted to this principle, in noticing the probability, that the larger and more distant planets, whose density is much less than that of the smaller and nearer ones, are in an earlier stage of condensation, and that the amount of heat still contained in their own masses may compensate in some degree for the deficiency of solar rays.

The history of the formation of the present crust of the earth, and of the introduction of living beings upon its surface, as revealed by the researches of modern geology, is then presented, in a series of masterly sketches of the condition of the globe and of its inhabitants at the principal periods. Many interesting, and, we believe, novel suggestions are offered in the course of this outline, which display the acuteness and fertility of the author's mind; but we must not stop to dwell upon them, as they have no direct bearing on the general argument. The following is the author's beautifully expressed summary:

"Thus concludes the wondrous chapter of the earth's history, which is told by geology. It takes up our globe at a period when its original incandescent state had nearly ceased; conducts it through what we have every reason to believe were vast, or at least very considerable, spaces of time, in the course of which many superficial changes took place, and vegetable and animal life was gradually developed; and drops it just at the point when man was apparently about to enter on the scene. The compilation of such a history from materials of so extraordinary a character, and the powerful nature of the evidence which these materials afford, are calculated to excite our admiration, and the result must be allowed to exalt the dignity of science, as a product of man's industry and his reason.

"If there is anything more than another impressed on our minds by the course of the geological history, it is, that the same laws and conditions of nature now apparent to us have existed throughout the whole time, though the operation of some of these laws may now be less conspicuous than in the early ages, from some of the conditions having come to a settlement and a close. That seas have flowed and ebbed, and winds disturbed their surfaces, in the time of the secondary rocks, we have proof on the yet preserved surfaces of the sands which constituted margins of the seas in those days. Even the fall of the wind—slanted rain is evidenced on the same tablets; the washing down of detached matter from elevated grounds which we see rivers constantly engaged in at the present time, and which is daily shallowing the seas adjacent to their mouths, only appear to have proceeded on a greater scale in earlier epochs. The volcanic subterranean force, which we see belching forth lavas on the sides of mountains, and throwing up new elevations by land and sea, was only more powerfully operative in distant ages. To turn to organic nature, vegetation seems to have proceeded then exactly as now. The very alternations of the seasons has been read in unmistakable characters, in sections of the trees of those days, precisely as it might be read in a section of a tree cut down yesterday. The system of prey amongst animals flourished throughout the whole of the prehuman period; and the adaptation of all plants and animals to their respective spheres of existence was as perfect in those early ages as it is still." (p. 147.)

After a few observations on the conditions which probably modified the operation of the more general laws at different periods, he thus continues:

"In pursuing the progress of development of both plants and animals upon the globe, we have seen an advance in both cases, along the line leading to the higher forms of organization. Amongst plants, we have first sea weeds, afterwards land plants; and amongst these, the simpler (cellular and cryptogamic) before the more complex. In the department of zoology, we see zoophytes, radiata, mollusca, articulata, existing for ages before there were any higher forms. The first step forward gives fishes, the humblest class of the vertebrata; and, moreover, the earliest fishes partake of the character of the next lowest sub-kingdom, the articulata. Afterwards come land animals, of which the first are reptiles, universally allowed to be the type next in advance from fishes, and to be connected with these by links of an insensible gradation. From reptiles we advance to birds, and thence to mammalia, which are commenced by marsupialia, acknowledgedly low forms in their class. That there is thus a progress of some kind, the most superficial glance at the geological history is sufficient to convince us. Indeed the doctrine of the gradation of animal forms has received a remarkable support from the discovery of this science, as several types formerly wanting to a completion of the series have been found in a fossil state.*

"It is scarcely less evident from the geological record, that the progress of organic life has observed some correspondence with the progress of physical conditions on the surface. We do not know for certain that the sea, at the time when it supported radiated, molluscous, and articulated families, was incapable of supporting fishes; but causes for such a limitation are far from inconceivable. The huge saurians appear to have been precisely adapted to the low muddy coasts and sea margins of the time when they flourished. Marsupials appear at the time when the surface was generally in that flat, imperfectly variegated state, in which we find Australia, the region where they now live in the greatest abundance, and one which has no higher native mammalian type. Finally, it was not till the land and sea had come into their present relations, and the former, in its principal continents, had acquired the irregularity of surface necessary for man, that man appeared. We have likewise seen reason for supposing that land animals could not have lived before the carbonigenous era, owing to the great charge of carbonic acid gas, presumed to have been contained in the atmosphere down to that time. The surplus of this having gone, as M. Brongniart suggests, to form the vegetation, whose ruins became coal, and the air being brought to its present state, land animals immediately appeared. So also sea plants were at first the only specimens of vegetation, because there appears to have been no place where other plants could be produced or supported. Land vegetation followed, at first simple, afterwards complex, probably in conformity with an advance of the conditions required by the higher class of plants. In short, we see everywhere throughout the geological history, strong traces of a parallel advance of the physical conditions and organic forms." (p. 150.)

Admitting, as we are ready to do, that the *general aspect* of the history bears out the inferences which our author (here following many previous speculators) founds upon it, we must stop to observe, that he has not exhibited his usual accuracy in regard to details. When these are closely examined, they are *not* found to support the hypothesis of progressive development,—i.e. of a regular sequence of forms, ascending in the scale of organization from the lower to the higher, in proportion to the advancing age of the earth. For, to advert to only a few cases,—in the older paleozoic, or protozoic rocks, we find, not merely zoophytes,

* Intervals in the series were numerous in the department of the pachydermata; many of these gaps are now filled up from the extinct genera found in the tertiary formation.

but, in association with them, mollusca and crustacea; and both the mollusca and crustacea were far from being the lowest of their kind. For of the remains of mollusca, those of cephalopods, the very highest of the group, form a proportion much greater than that, which the existing species of that class bear to the inferior molluscs. And although the exact zoological place of the trilobites, which are so abundant in the paleozoic rocks, cannot be determined, there can be no doubt whatever that they were by no means the lowest of the crustaceous series. Again, although it is quite true that the earliest fishes, whose remains we find, present many points of approximation to the invertebrata, yet the *next*, those belonging to the *sauroid* family, must be considered as bearing an equally close affinity to the class of reptiles. Our author has been misled by the cartilaginous condition of the skeleton which prevails in that group, to speak of them as an order of "mean organization," (p. 63;) but this is by no means the case, since the existing sauroid fishes are superior in almost every particular, except the imperfect ossification of the skeletons, to all others of the class,—being especially remarkable for the development of their nervous and muscular systems, and for the advance in the mode of their reproduction towards the true viviparous type. We might adduce many other facts to the same effect; which seem to us to militate strongly against the deduction of any general law of progressive development from geological phenomena. Whilst objecting, however, to the *form* of our author's hypothesis of the origin of organized beings, we do in effect assent to his fundamental ideas; which we shall hereafter endeavour to present under a more satisfactory aspect. These ideas are so well expressed in the following quotations, that we shall not attempt to add or to qualify them.

"There are, indeed, abundant appearances, as if, throughout all the changes of the surface, the various kinds of organic life invariably *pressed in*, immediately on the specially suitable conditions arising; so that no place which could support any form of organic being, might be left for any length of time unoccupied. Nor is it less remarkable how various species are withdrawn from the earth, when the proper conditions for their particular existence are changed. The trilobite, of which fifty species existed during the earlier formations, was extirpated before the secondary had commenced, and appeared no more. The ammonite does not appear above the chalk. The species and even genera of all the early radiata and molluscs were exchanged for others long ago. Not one species of any creature which flourished before the tertiary (Ehrenberg's infusoria excepted), now exists; and of the mammalia, which arose during that series, many forms are altogether gone, while of others we have now only kindred species. Thus, to find not only frequent additions to the previously-existing forms, but frequent withdrawal of forms which had apparently become inappropriate—a constant shifting as well as advance—is a fact calculated very forcibly to arrest attention.

"A candid consideration of all these circumstances can scarcely fail to introduce into our minds a somewhat different idea of organic creation from what has hitherto been generally entertained. That God created animated beings, as well as the terraqueous theatre of their being, is a fact so powerfully evidenced and so universally received, that I at once take it for granted. But in the particulars of this so highly supported idea we surely here see cause for some reconsideration. It may now be inquired, In what way was the creation of animated beings effected? The ordinary notion may, I think, be not unjustly described as this—that the Almighty Author produced the progenitors of all the existing species by some sort of personal or immediate exertion. But how does this notion comport with what we have seen of the gradual advance of species, from the humblest to the highest? How can we suppose an immediate exertion of this creative power at one time to produce zoophytes, another time to add a few marine molluscs, another to bring in one or two conchifera, again to produce crustaceous fishes, again perfect fishes, and so on to the end? This would surely be to take a very mean view of the creative power; to, in short, anthropomorphize it, or reduce it to some such character as that borne by the ordinary proceedings of mankind. And yet this would be unavoidable; for that the organic creation was thus progressive through a long space of time, rests on evidence which nothing can overturn or gainsay. Some other idea must then be come to with regard to *the mode* in which the Divine Author proceeded in the organic creation. Let us seek in the history of the earth's creation for a new suggestion on this point. We have seen powerful evidence that the construction of this globe and its associates, and, inferentially, that of all the other globes of space, was the result, not of any immediate or personal exertion of the Deity, but of natural laws which are expressions of His will. What is to hinder our supposing that the organic creation is also a result of natural laws which are, in like manner, an expression of His will? More than this, the fact of the cosmical arrangements being an effect of natural law, is a powerful argument for the organic arrangements being so likewise; for how can we suppose that the august Being who brought all these countless worlds into form by the simple establishment of a natural principle, flowing from his mind, was to interfere personally and specially on every occasion when a new shell-fish or reptile was to be ushered into existence on *one* of these worlds? Surely this idea is too ridiculous to be for a moment entertained." (pp. 151-4.)

The complete accordance of these views, with those some time ago propounded by ourselves (vol. V, p. 342), must be evident, we think, to our readers. To the objection which some timid religionists may urge against them, that they are inconsistent with the Mosaic record, we simply reply, with our author, that we do not think it right to adduce that record either in support of, or in objection to, any scientific hypothesis, based upon the phenomena of nature; and this for many reasons, but particularly for this, that there is not the least appearance of an intention in that book, or in any other part of scripture, to give philosophically exact views of the divine operations in the universe at large, or to do anything else than exhibit the Deity in his relations to man. The views just expressed are certainly not *more* inconsistent with the Mosaic account of the *manner* of creation, than are the more detailed conclusions of geologists in regard to its *history*;—perhaps not so much so.

The following passage accords so remarkably with our own views, as stated in a former page of this article, that it might appear a needless repetition to quote it; but the ideas which it presents are so felicitously expressed, that we cannot forbear introducing it.

"To a reasonable mind the Divine attributes must appear, not diminished or reduced in any way by supposing a creation by law, but infinitely exalted. It is the narrowest of all views of the Deity, and characteristic of a humble class of intellects, to suppose him acting constantly in particular ways for particular occasions. It, for one thing, greatly detracts from his foresight, the most undeniable of all the attributes of Omnipotence. It lowers him towards the level of our own humble intellects. Much more worthy of him it surely is, to suppose that all things have been commissioned by him from the first, though neither is he absent from a particle of the current of natural affairs, in one sense, seeing that the whole system is continually supported by his providence. Even in human affairs, if I may be allowed to adopt a familiar illustration, there is a constant progress from specific action for particular occasions, to arrangements which, once established, shall continue to answer for a great multitude of occasions. Such plans the enlightened readily form for themselves, and conceive as being adopted by all who have to attend to a multitude of affairs, while the ignorant suppose every act of the greatest public functionary to be the result of some special consideration and care on his part alone. Are we to suppose the Deity adopting plans which harmonize only with the modes of procedure of the less enlightened of our race? Those who would object to the hypothesis of the creation by the intervention of law, do not perhaps consider how powerful an argument in favour of the existence of God is lost by rejecting this doctrine. When all is seen to be the result of law, the idea of an Almighty Author becomes irresistible, for the creation of a law for an endless series of phenomena—an act of intelligence above all else that we can conceive—could have no other imaginable source, and tells, moreover, as powerfully for a sustaining as for an originating power." (p. 158.)

The author then passes from general to *particular considerations respecting the origin of the animated tribes;* and thus introduces the subject: "The general likelihood of an organic creation by law having been shown, we are next to inquire if science has any facts tending to bring the assumption more nearly home to nature. Such facts there certainly are; but it cannot be surprising that they are comparatively few and scattered, when we consider that the inquiry is into one of nature's profoundest mysteries, and one which has hitherto engaged no direct attention in almost any quarter." (p. 165.) It is the object of this chapter to bring together the known facts upon this subject; and we are sorry to be obliged to remark, that there is such a looseness in the author's statements, and so much disposition to interpret phenomena in accordance with his hypothesis, that we regret its presence in the book. It is in his reasoning upon the general question, that we recognize the mind of the true philosopher. The sublime inductions of astronomy, and the revelations of geological history, have never, perhaps, been so well interpreted with reference to the attributes of the Creator. But when the author comes to analyse the difficult problems of physiology, we see the workings of a mind better fitted (like that of the immortal Bacon) to deal with the *general* than the *particular*,—with *reasoning*, than with *facts.* The following is a specimen of the fallacies, which we feel called upon to notice with regret. After adverting to the discoveries of modern science in regard to the production and multiplication of *cells*, and rightly asserting that "all animated nature may be based on this mode of origin," he thus proceeds:—"*The fundamental form of organic being is a globule, having a new globule forming within itself*, by which it is in time discharged, and which is again followed by another and another in endless succession." Now, on a wrong application of a single term, a whole fabric of error may be erected; and we stop, therefore, to point out the author's confusion between a living *cell*, in itself an organized active being, capable of absorbing, respiring, secreting, and reproducing its kind,—and a mere *globule*, characterized only by its form, and having no vital actions whatever, (for the reproduction of *glo-ules* is a fallacy).

"It is of course obvious," continues our author, "that if these globules could be produced, by any process, from inorganic elements, we should be entitled to say, that the fact of a transit from the inorganic into the organic had been witnessed in that instance; the possibility of the commencement of animated creation by the ordinary laws of nature, might be considered as established. Now, it was given out some years ago by a French physiologist [Dutrochet], *that globules could be produced in albumen by electricity.* If, therefore, these globules be identical with the cells which are now held to be reproductive, it might be said that the production of albumen by artificial means is the only step in the process wanting. This has not yet been effected; but it is known to be only a chemical process, the mode of which may be any day discovered in the laboratory; and two compounds, perfectly coordinate, urea and allantoin, have actually been produced." (p. 173.)

So that, in our author's opinion, we have only to manufacture albumen, to be able to produce "reproductive globules," out of inorganic matter, to an unlimited extent. The world is not, however, quite prepared for this announcement. The *globules* produced in albumen by the agency of electricity are far from being *living cells;* and no albumenous matter can exhibit vital actions,—so far as our present experience enables us to determine,—without having been subjected to the action of a previously-existing organism. Hence, even supposing that we could *manufacture* albumen (an idea we have long entertained as possible), we should be very little nearer to the manufacture of a reproductive cell, which no one has ever yet generated out of albumen ready prepared to his hand. We must imagine the author to be practically unacquainted with the matter in question; or he would not have fallen into the grievous error of confusing a cell with a globule.—So, also, in regard to the artificial production of a substance resembling shell, we apprehend that the results of recent microscopical inquiries are completely opposed to the idea of any real conformity between the two substances; the corespondence between their optical phenomena being no greater, than that which can be produced by impressing a series of lines, of sufficient delicacy and proximity to give the iridescent lustre, on *any* hard and polished surface. The author, however, does not lay much stress on these incidents; and thus proceeds to inquire into what actual experience says regarding the origin of life.

"Are there, it will be said, any authentic instances of either plants or animals, of however humble and simple a kind, having come into existence otherwise than in the ordinary way of generation, since the time of which geology forms the record? I may be answered, that the negative of this question could not be by any means formidable to the doctrine of law-creation, seeing that the conditions necessary for the operation of the supposed life-creating laws may not have existed within record to any great extent. On the other hand, as we still see the physical laws of early times still acting with more or less force, it might not be unreasonable to expect that we should still see some remnants or partial and occasional workings of the life-creating energy amidst a system of things generally stable and at rest. Are there, then, any such remnants to be traced in our own day, or during man's existence upon earth. If there be, it clearly would form strong evidence in favour of the doctrine, as what now takes place upon a confined scale, and in a comparatively casual manner may have formerly taken place on a great scale, and as the proper and eternity-destined means of supplying a vacant globe with suitable tenants. It will at the same time be observed, that the earth being now supplied with both kinds of tenants in great abundance, we only could expect to find the life-originating power at work, in some very special and extraordinary circumstances, and probably only in the inferior and obscurer departments of the vegetable and animal kingdoms." (pp. 175-6.)

This we consider to be very fair argument; and we cannot but regret to find that the author has not brought any additional evidence to bear upon the question. He quotes the opinions of various writers in regard to the production of infusoria, entozoa, &c.; and points out certain obvious fallacies in the reasoning, by which the idea of their uniform origin

*ab ovo* is usually supported. Thus it has been shown that, when a vegetable infusion is debarred from the contact of the atmosphere, by being closely sealed up or covered with a layer of oil, no animalcules are produced; but it may be said, on the other hand, that the exclusion of the air may prevent some simple condition necessary for the aboriginal development of life. Let the experiment be tried in a different manner,—for instance, by admitting air that had been passed through a tube sufficiently hot to destroy any organic germs it might contain; and the appearance of animalcules in the infusion, under such circumstances, would doubtless be a much stronger argument in favour of their spontaneous production. But it would be far from an unanswerable one; since it might fairly be objected, that ova were not destroyed by the heat, or that the tissues of the plant had contained the parent animalcules. This last objection must be admitted to have great weight, when it is remembered that the residence of animalcules in living plants is by no means uncommon; as has been shown in regard to certain Rotifera, which inhabit the perforated cells of the sphagnum, and the Vibriones which are so well known to frequent the grains of wheat. It must be admitted, we think, that *our general experience* is in favour of reproduction by *germs of some kind,* (to what extent *similar* germs may produce *different* forms, according to the circumstances of their development, we shall presently inquire;) and therefore we feel a right to explain away phenomena which apparently require a different interpretation, in any mode not free from insuperable objections; in other words, we cannot admit the truth of the doctrine of spontaneous generation, until a living being has been produced under circumstances which render the pre-existence of a germ *impossible.*

Such a case our author thinks is presented by the production of the celebrated *Acarus Crossii*, which was first noticed in a solution of silicate of potass, when submitted to slow galvanic action, for the purpose of obtaining crystals of silex. We have Mr. Crosse's own authority for the statement, that he never made any experiments for the purpose of producing those creatures; but that they presented themselves in the course of experiments devised for entirely different purposes. By Mr. Weekes of Sandwich, however, the experiments were repeated and varied with this express object, and with similar results; a larger number of acari being generated, when ferrocyanate of potass was the compound employed,—which is imputed by our author to the carbon which it includes. We are sorry to notice another error of detail in the account given by the author of these experiments. Quoting Mr. Weekes's statement that, when the silicate of potass was galvanized, it first became turbid, and then of a milky appearance, and that round the negative wire of the battery, there gathered a quantity of *gelatinous matter,* he jumps to the conclusion that this consists of the organic compound, or proximate principle, *gelatin,* of which there is not the shadow of a proof. Let it be remembered that the silicate of potass, or liquor of flints, has itself a very *gelatinous appearance;* but we might as well set down its subsequent *milky aspect* to the production of milk, as conclude, without further proof, that the gelatinous aspect is due to the presence of gelatin. Putting aside, however, all that is questionable, and confining ourselves to ascertained facts, we believe that we are justified in stating, that none of the easy solutions, which have been offered of the difficult problem presented by the appearance of this acarus can be admitted. In Mr. Weekes's experiments, every precaution *seems* to have been taken to avoid fallacy. The solid materials employed were submitted to a heat sufficient to calcine them; the fluids were distilled; and the galvanic action was conducted within a vessel, from the interior of which the air was completely excluded, and under oxygen which had been disengaged from manganese at red heat. It is not a little remarkable, that *the same species* of animal, and no other, should present itself under these circumstances, as in Mr. Crosse's experiments. Not the least curious part of its history, is the series of metamorphoses which it undergoes before quitting the solution; these being entirely different from the very slight changes which other acari undergo after their emersion from the egg. Further, we believe it may be positively asserted, that, in whatever mode these acari are first generated, *it is not from eggs;* since, after they have escaped from the solùtion, they live in the neighbourhood, and readily breed; and their eggs, which we have ourselves seen, are quite large enough to have been readily visible in the solution, had they existed there. Not entertaining the opinion, which a certain clergyman is reported to have expressed of Mr. Weekes's investigations,—that "it was a very dark business, and such as no christian man ought to engage in,"—we please ourselves with the hope of being able to throw some light, ere long, upon the history of this interesting creature, by the careful study of the physiological peculiarities of its development; leaving it to those eminent in physical science to investigate the physical conditions under which it is produced. Should the author's views be realized, the *acarus Crossii* will indeed be worthy to take rank aso ne of the most—if not *the* most—physiologically-important species of the whole animal creation; affording proof of the *possible* origin of living beings of complex structure and varied actions, from the combination of particles of inorganic matter under peculiar conditions; and thus outshining, in the eyes of the physiologist, the transcendant merits of frogs, rabbits, dogs, asses, &c. as subjects of experiment. But before we can yield our belief to so wonderful an event, we must be much more assured than we are, that the production of the animal, in both Crosse's and Weekes's experiments, was *impossible* in the ordinary way of generation.

Let us now see how our author meets the objection, which will be most certainly raised against his inferences from these experiments:—as, indeed, it has already been, in regard to the interpretation supposed (but erroneously) to have been offered by Mr. Crosse himself.* It has been held to be impious, even to surmise that animals could have been formed through any instrumentality of an apparatus devised by human skill. To this it may be replied:

"The supposition of impiety arises from an entire misconception of what is implied by an aboriginal creation of insects. The experimentalist could never be considered as the author of the existence of these creatures, except by the most unreasoning ignorance. The utmost that can be claimed for, or imputed to him is, that he arranged the natural conditions under which the true creative energy,

* Mr. Crosse has uniformly declined expressing an opinion upon the subject; wisely restrictiug himself to a statement of the facts witnessed by himself.

that of the Divine Author of all things, was pleased to work in that instance. On the hypothesis here brought forward, the *Acarus Crossii* was a type of being ordained from the beginning, and destined to be realized under certain physical conditions. When a human hand brought these conditions into the proper arrangement, it did an act akin to hundreds of familiar ones which we execute every day, and which are followed by natural results; but it did nothing more. The production of the insect, if it did take place as assumed, was as clearly an act of the Almighty himself, as if he had fashioned it with his hands." (pp. 188-9.)

Let us now briefly recapitulate the ground over which we have passed. We have endeavoured, with our author's aid to show in the *first* place, that all our knowledge of the attributes of the Deity, derived both from the philosophic study of his operations in the physical universe, and from revelation itself, leads to the belief in the uniformity of his plan of action, from the beginning of the existing system of things to the present day; or in other words, that the laws he at first laid down for himself, have not been modified by any subsequent contingencies.

*Secondly*, we have seen that both astronomy and geology combine in representing our globe, as having passed through a series of phases; commencing perhaps with the gradual condensation of a diffused nebular matter, but at any rate with the gradual cooling of a liquid mass in a state of incandescence *

*Thirdly*, that organic life could not have appeared, until after a *crust* had been formed on the surface of the globe, and this crust had been covered with water; all which must have occupied a vast period of time.

*Fourthly*, that subsequently to the first appearance of organic life upon the globe, its forms have been continually undergoing change;—some becoming extinct, owing to a change of the conditions required for their existence;—whilst others have been introduced, in conformity with the new circumstances which have arisen from time to time.

*Fifthly*, that to reconcile these successive changes in the organic forms existing on the earth, with that idea of unvarying law, which we regard as essential to our conception of the Deity, we must believe all these forms as having been previously arranged or determined by Him, in accordance with the conditions which would successively arise; and to have come into existence, as soon as these conditions presented themselves, without any *fresh* act of creative power, or any special interference. This view, we may remark, derives remarkable confirmation from the fact of which the philosophic naturalist is now well aware, that his classification can only be complete, when it embraces *all* the fossil as well as the existing forms of plants and animals; the hiatuses existing among the latter, which are most perplexing to the systematist, and which prevent him from obtaining even a glimpse of the *plan* on which the organized creation has been arranged, being filled up, often in the most remarkable and unexpected manner, by the former.

*Sixthly*, that, with reference to the creative *nisus* of the Deity, which (as it has been seen) we most fully recognize in *all* cases, it cannot be regarded as essentially different, whether He constructed the new forms of organized beings out of the materials supplied by the inorganic world,

* This last view is supported by the fact of the *spheroidal* form of the earth, and by the still more spheroidal form of Jupiter; the excess of the equatorial over the polar diameter being, in each case, exactly what would result from centrifugal force acting on *liquid masses* rotating at the present diurnal rates of these two planets respectively.

*at each period* that such an interposition was rendered necessary by a change of condition: or whether He *originally* endowed the particles of what we commonly term inorganic matter with the power of *themselves combining* into these new forms, whenever the requisite conditions should arise. As in the case of the calculating machine, however, it gives a much higher notion of the character of the designing mind, to regard these operations as the working out of the original plan, than to attribute them to special and repeated interpositions.

*Seventhly*, that our limited experience must not be taken as affording a sufficient demonstration of the falsity of this position, even though we should not be able to produce a single case in which a new form of organic life has been thus developed under our own observation; and that it is, to say the least, an undecided question, whether this has not actually taken place.

It may serve to add probability to these views, if we turn our attention to that which must necessarily be admitted, on *any* hypothesis, of all those forms of matter which are employed in the construction of a living being. We see a simple germ—the nucleus of a cell—develope itself into a feeling, moving, thinking Man, by drawing into itself and combining into new forms the particles of what we are accustomed to call Inorganic matter. These new forms are caused, by the very act of combination, to manifest properties of a new and peculiar kind; and their actions constitute the life of the being. Hence we must attribute to all those substances, which are thus drawn from the inorganic into the organic mode of existence, *a latent capacity* for the latter;—just as we say that the oxygen, hydrogen, carbon, and nitrogen, which make up the organic substance termed muscular fibre, and which in *that* state or mode of combination possess certain *vital* properties, possess also *a latent capacity* for combining in that mode of aggregation termed crystalline, and for exhibiting the solubility, translucency, and other qualities of a salt (all of which are totally opposed to its vital properties, and cannot co-exist with them), when united into the form of cyanate of ammonia. If we were *only* acquainted with these elements as they exist in organic compounds, their transposition into a crystalline salt would be almost as marvellous to us as the opposite change is now. If this *latent organizability or vitality* be admitted (as we conceive logical proof to have been given that it must), as a property of a large proportion of what we call inorganic matter, is there any such wonderful difficulty in imagining that it may be brought into play in some other manner than by the agency of a pre-existing germ? We think not. But let further investigation and more extended experience decide.

Thus far we have kept in perfect agreement with our author, on all main questions; though we are not quite so ready as himself to admit, as *proved*, what is certainly very far from being demonstrated. And though we have expressed ourselves somewhat strongly in favour of what may be termed the *à priori* argument, drawn from the attributes of the Deity, as made known to us by his works and by his Word, yet we would endeavour to guard ourselves and others from the presumption of supposing that, in the absence of any positive data on the subject, any such argument can be sufficient to decide the question—presuming, as it does, that the *whole* Mind of the Deity, on this subject, is known to *us*, his feeble imperfect creatures. Our aim has rather been, to set before our readers that which we think *may be* believed, than to dictate to them what *is to be* believed,—to remove the errors and prejudices connected with hitherto-received modes of thought—rather than to establish any new system.

In the remaining part of our author's scheme, however, we cannot so fully accord with him. This is contained in a chapter entitled "Hypothesis of the Development of the Vegetable and Animal Kingdoms;" and it will be understood from the following extracts. After directing attention to the fact, that, notwithstanding the almost countless varieties of form and structure presented by organized beings, there are fundamental resemblances, which enable us to group them round particular types;—and, after noticing some of the modifications which the same organs undergo in different species, for the purpose of adapting them to different conditions of existence, he thus continues:

"In such circumstances, a candid mind which sees nothing impious or unphilosophical in the idea of a new creation, will be disposed to think, that there is less difficulty in believing in such a creation having actually taken place, than in believing that in two instances, separated in place and time, exactly the same insects should have chanced to arise from concealed ova, and these heretofore unknown." (p. 190.)

"These facts clearly show how all the various organic forms of our world are bound up in one,—how a fundamental unity pervades and embraces them all, collecting them, from the humblest lichen up to the highest mammifer, in one system, the whole creation of which must have depended upon one law or decree of the Almighty, though it did not all come forth at one time. After what we have seen, the idea of a separate exertion for each must appear totally inadmissible. The single fact of abortive or rudimentary organs condemns it, for these, on such a supposition, could be regarded in no other light than as blemishes or blunders, the thing of all others most irreconcilable with that idea of Almighty Perfection, which a general view of nature so irresistibly conveys. On the other hand, when the organic creation is admitted to have been effected by a general law, we see nothing in these abortive parts but harmless peculiarities of development, and interesting evidences of the manner in which the Divine Author has been pleased to work." (p. 197.)

He then dwells much on the modern physiological doctrine of progressive development, as occurring in every one of the higher animals; and adverts to some curious analogies between the fœtal conditions of existing animals, and the earliest forms of similar animals whose traces can be detected. These analogies, however, do not by any means appear to us sufficient to demonstrate, although they may render probable, "the parity, or rather identity, of laws presiding over the development of the animated tribes on the face of the earth, and that of the individual in embryo." On this principle, his hypothesis of the progressive changes in the fauna and flora of our own and other globes, is erected—after the following fashion:

"The tendency of all these illustrations is to make us look to *development* as the principle which has been immediately concerned in the peopling of this globe, a process extending over a vast space of time, but which is nevertheless connected in character with the briefer process by which an individual being is evoked from a simple germ. What mystery is there here, and how shall I proceed to enunciate the conception which I have ventured to form of what may prove to be its proper solution! It is an idea by no means calculated to impress by its greatness, or to puzzle by its profoundness. It is an idea more marked by simplicity, than perhaps any other of those which have explained the great secrets of nature. But in this lies perhaps one of its strongest claims to the faith of mankind.

The whole train of animated beings, from the simplest and oldest up to the highest and most recent, are, then, to be regarded as a series of *advances of the principle of development*, which have depended upon external physical circumstances to which the resulting animals are appropriate. I contemplate the whole phenomena as having been in the first place arranged in the counsels of Divine Wisdom, to take place, not only upon this sphere, but upon all the others in space, under necessary modifications, and as being carried on from first to last, here and elsewhere, under immediate favour of the creative will or energy. The nucleated vesicle, the fundamental form of all organization we must regard as the meeting-point between the inorganic and the organic, the end of the mineral and beginning of the vegetable and animal kingdoms, which thence start in different directions, but in perfect parallelism and analogy." (p. 202-4.)

In regard to this analogy he elsewhere remarks (p. 237), that "so complete does it appear, even in the present imperfect state of science, that I fully expect in a few years to see the animal and vegetable kingdoms duly ranked up against each other in a system of parallels, which will admit of our assigning to each species in the former the particular shrub or tree corresponding to it in the latter, all marked by unmistakeable analogies of the most interesting kind." Disposed as we are to seek for and recognize these analogies (see vol. I, p. 1, *et seq.*), we cannot feel nearly so sanguine as our author, in regard to the extent to which they are likely to be pushed; since it is only in a most general and comprehensive view of the principal groups of the two kingdoms respectively, that any analogy can be detected between them; and the entire difference in the *fundamental plan* of the two grand divisions of organized beings, and, consequently, in all the details of their structure and economy, entirely forbid the idea, in our apprehension, that any such individual resemblance can exist. But we return from this digression to our author's hypothesis, which will perhaps be best understood by an extract from a later part of the chapter:

"The idea then which I form of the progress of organic life upon the globe, and the hypothesis is applicable to all similar theatres of vital being, is *that the simplest and most primitive type, under a law to which that of like production is subordinate, gave birth to the type next above it, that this again produced the next higher, and so on to the very highest*, the stages of advance being in all cases very small, viz. from one species only to another; so that the phenomenon has always been of a simple and modest character. Whether the whole of any species was at once translated forward, or only a few parents were employed to give birth to the new type, must remain undetermined; but supposing that the former was the case, we must presume that the moves along the line or lines were simultaneous, so that the space vacated by one species was immediately taken by the next in succession, and so on back to the first, for the supply of which the formation of a new germinal vesicle out of inorganic matter was alone necessary. Thus the production of new forms, as shown in the pages of the geological record, has never been anything more than a new stage of progress in gestation, an event as simply natural, and attended as little by any circumstance of a wonderful or startling kind, as the silent advance of an ordinary mother from one week to another of her pregnancy. Yet, be it remembered, the whole phenomena are, in another point of view, wonders of the highest kind, for on each of them we have to trace the effect of an Almighty will which had arranged the whole in such harmony with external physical circumstances, that both were developed in parallel steps, and probably this development upon our planet is but a sample of what has taken place, through the same cause, in all the other countless theatres of being which are suspended in space." (pp. 222-3.)

We shall leave him to explain, also, the difference between *his* hypothesis and that of Lamarck; which has been most deservedly ridiculed by almost every writer who has alluded to it:

"Early in this century, M. Lamarck, a naturalist of the highest character, suggested an hypothesis which deservedly incurred much ridicule, although it contained a glimmer of the truth. He surmised and endeavoured, with a great deal of ingenuity, to prove that one being advanced in course of generations to another, in consequence merely of its experience of wants calling for the exercise of its faculties in a particular direction, by which exercise new developments of organs took place, ending in variations sufficient to constitute a new species. Thus, he thought that a bird would be driven by necessity to seek its food in the water, and that, in its efforts to swim, the outstretching of its claws would lead to the expansion of the intermediate membranes, and it would thus become web-footed. Now, it is possible that wants and exercise of faculties have entered in some manner into the production of the phenomena which we have been considering; but certainly not in the way suggested by Lamarck, whose whole notion is obviously so inadequate to account for the rise of the organic kingdoms, that we only can place it with pity among the follies of the wise. Had the laws of organic development been known in his time, his theory might have been of a more imposing kind. It is upon these that the present hypothesis is mainly founded. I take existing natural means and show them to have been capable of producing all the existing organisms, with the simple and easily-conceivable aid of a higher generative law, which we perhaps still see operating upon a limited scale. I also go beyond the French philosopher to a very important point, the original Divine conception of all the forms of being which these natural laws were only instruments in working out and realizing." (pp. 230-1.)

It is admitted that there is no satisfactory proof of the development, under our observation, of any new race of animals, differing from its progenitors in a mode sufficiently marked to constitute that which a naturalist would regard a distinct *species.* On this word *species* we must for a moment dwell, in order to explain the sense in which we use it. Two races of animals are said to be of *the same species*, if they are not distinguished from each other by any peculiarities, but such as the one may be supposed to have lost, or the other to have gained, with the lapse of time or variations in external circumstances. When this is the case, it is presumed that these two races had, or may have had, *common progenitors.* On the other hand, when a *constant* difference exists between two races, such as we cannot account for in the ways just mentioned, we believe that they must have been *originally distinct*, or have been of *different species.* It is well known that there are some races, as those of the dog, amongst which the differences are very considerable; yet there is reason to regard them all as descendants of one stock, and many zoologists believe that stock to have been the wolf. Still, amongst all these, there is an exact agreement in those characters which are elsewhere found best to serve the purposes of classification; viz. those derived from the form of the teeth and extremities: and there is a tendency among them all to return to a common type, when the races are no longer kept distinct by the intervention of man. But among the feline races, the case is very different; for the several species of that genus—agreeing so closely in osteology that, except in point of size, there is no characteristic distinction between their respective skeletons—are found to be sufficiently distinguished from each other by the markings upon their skins—characters which are in *other* cases subject to extreme uncertainty, but which are *here* so constant as to present scarcely the slightest variation amongst different individuals. Thus, if a certain patch or stripe be repeated from generation to generation with *certainty*, in a feline race, the naturalist regards this a sufficient proof of the specific difference of that race from another that is differently marked. The domestic cat is the only one of the group that is liable to any considerable variation ; and in *this* species, as every one knows, the markings characteristic of the several breeds or races are *not* constant, and are therefore not indicative of original difference. This striking contrast between *variable* and *invariable* groups of animals nearly allied to each other, is found through the whole kingdom ; every division of it containing *some* species, which do *not* change their forms or other characteristics under *any* circumstances, but which cease to exist if a change in their conditions renders these unsuitable ; whilst it also includes others in whose physical and psychical constitutions there is such a susceptibility of undergoing modification, that new forms and new instincts may arise, and thus new and very different races may be originated.

Now, it is evident that the *known invariability* of a large proportion of the species composing the animal kingdom, is a powerful objection to the hypothesis in question ; still it must be admitted to be not an unanswerable argument, since it may be said that we have not had them sufficiently long under our observation, nor seen them placed in a sufficient variety of circumstances, to enable us to assert their unalterability so dogmatically. Still, it is evidently for those who deny what all experience teaches, to adduce some proof of their position ; and here we think that our author fails. The *known variability* of certain other species does not much help his doctrine ; for though there are many breeds of dogs and horses, sheep and oxen, they are *all* dogs and horses, sheep and oxen ; differing in subordinate peculiarities, but in no instance becoming elevated towards any higher form, or acquiring any entirely new faculty. It has been, we think, completely demonstrated, that, in all known instances in which any *acquired* peculiarity is transmitted from parents to offspring, it has reference to the *original* habits of the race. Thus, particular breeds of dogs are distinguished by peculiarities of instinct, as well as of conformation ; but all these peculiarities have reference to their natural habits, and may be regarded as resulting from a higher development of some particular pre-existing tendencies, others remaining in consequence almost rudimentary. We might, doubtless, produce the same results by training particular races of men ; indeed, we may even now witness them, by comparing any two races, which, having long existed under opposite conditions, have had a certain physical and mental constitution impressed upon them in consequence, but are still *men*.

We do not regard our author as at all more fortunate in the illustrations he has attempted to draw from the study of development. For neither in the case of the butterfly which comes forth in the condition of a worm, the frog which begins its *extra-oval* life in the condition of a fish, or the queen-bee which is produced by the increased development of a neuter, do we recognize any indication whatever of an advance upon the type of the species. Of the two former cases the history is simply this,—that, for certain purposes, which it is not difficult to trace, the embryo comes forth, at an unusually early period, in possession of a set of organs which enable it to obtain food for itself; and thus to support *itself* during the remaining period of its development, instead of being dependent upon the nutriment supplied by the parent. The larva of an insect (we speak of those undergoing a *complete* metamorphosis) is still as much an embryo, in regard to the condition of its tissues, as if it were being developed, like that of a spider or acarus, within its egg; and in its pupa state—a period of *organic development* and of *dormant animality*—we recognize a return to the passive condition of the embryo of other animals, preparatory to the final emersion of the imago in the fully-developed condition. The recent direction of zoological research towards the natural history of the lower tribes of animals, has abundantly shown that the phenomenon of metamorphosis, which is rather the *exception* in the higher classes, is the *rule* in the lower; and that it is, in all cases of the same character; viz. a temporary assumption by the *embryo*, of a condition analogous to that of some of the *lower* tribes, enabling it to obtain its own food, and consequently to acquire the materials for its further development without the assistance of its parent. Such facts, it will be obvious, afford no support whatever to the idea, that a true worm can ever become a butterfly, or a true fish become a frog.—The case of the queen-bee is somewhat different, though still analogous. It was ascertained by Huber that the larva of the working-bee may be developed, by a particular mode of treatment, into a queen; but this simply results from the evolution of the sexual organs, which, on the ordinary regimen of the workers, remain atrophied. Hence every worker may be considered as an undeveloped queen, in regard to the sexual apparatus and its connected instincts, these characteristics of the latter being rudimentary in the former; but, on the other hand, every queen may be regarded as an undeveloped worker, in regard to certain other organs (the instruments of its destined labours), and the instincts connected with them, which are developed in the latter but not in the former. In other words, all that this fact proves is, that the same germ may be developed into two *different* forms, according to the circumstances of its early growth; it by no means demonstrates any capability of advance in the race at large. If the queen-bee, thus produced from a worker-larva, were to produce only a royal race, equally gifted with herself, we might admit the validity of the argument; but, instead of this being the case, she becomes, like her sister sovereigns, the parent of as rank a democracy as ever flourished; and the new and improved edition is as far as ever from blessing our sight. Moreover, it is by no means proved, that the queen is more elevated in type than the workers; for it is a mere assumption that she *directs* the labours of the hive. *Her* province is simply to increase the population; whilst the offices of the workers are far more varied, and their psychical character appears to be higher.—The study of the physiology of plants, and of that of the lower tribes of animals, affords numerous instances of a similar character; where *like* germs are developed, under varying circumstances, into *unlike* beings; but in all these cases, there are certain limitations to the varieties of form which are produced, and we cannot say that either of them is a decided advance upon another.

We have dwelt upon this point the longer, because our author's arguments have a certain speciousness about them, which does not allow their true value to be estimated, until they have been carefully sifted. The progressive development of species on the surface of the globe, and the successive appearance of different kinds of animalcules in an infusion of decaying matter, are also referred to as confirmations of the doctrine which he advances; but we have already shown, that the detailed study of palæontology does not bear out the inferences suggested by a cursory view of it; and the hypothesis we are about to suggest will be found, we think, much more conformable to the facts supplied by geological history, as well as to the second of the cases just noticed. We must first quote, however, on account of the just and beautiful spirit which pervades it, a passage, in which our author combats the objections he anticipates to that account of the origin of the human race, which his theory involves:

"But the idea that any of the lower animals have been concerned in any way with the origin of man—is not this degrading? Degrading is a term expressive of a notion of the human mind; and the human mind is liable to prejudices which prevent its notions from being invariably correct. Were we acquainted for the first time with the circumstances attending the production of an individual of our race, we might equally think them degrading, and be eager to deny them, and exclude them from the admitted truths of nature. Knowing this fact familiarly and beyond contradiction, a healthy and natural mind finds no difficulty in regarding it complacently. Creative Providence has been pleased to order that it must be so, and it must therefore be submitted to. Now, the idea as to the progress of organic creation, if we become satisfied of its truth, ought to be received precisely in this spirit. It has pleased Providence to arrange that one species should give birth to another, until the second highest gave birth to man, who is the very highest; be it so, it is our part to admire and to submit. The very faintest notion of there being anything ridiculous or degrading in the theory—how absurd does it appear when we remember that every individual amongst us actually passes through the characters of the insect, the fish, and reptile (to speak nothing of others), before he is permitted to breathe the breath of life. But such notions are mere emanations of false pride and ignorant prejudice. He who conceives them little reflects that they in reality involve the principle of a contempt for the works and ways of God. For it may be asked, if He, as appears, has chosen to employ inferior organisms as a generative medium for the production of higher ones, even including ourselves, what right have we, his humble creatures, to find fault? There is also in this prejudice an element of unkindliness towards the lower animals, which is utterly out of place. These creatures are all of them part products of the Almighty conception, as well as ourselves. All of them display wondrous evidence of his wisdom and benevolence. All of them have had assigned to them by their Great Father a part in the drama of the organic world, as well as ourselves. Why should they be held in such contempt? Let us regard them in a proper spirit, as parts of the grand plan, instead of contemplating them in the light of frivolous prejudices, and we shall be altogether at a loss to see how there could be any degradation in the idea of our race having been genealogically connected with them." (p. 233-5.)

We shall now briefly indicate the view which we would substitute for the author's, in regard to the development of the several varieties of animal and vegetable life, which have existed, or which now exist upon the globe. We would premise, however, that we by no means desire to advance it as a formal theory, or as one entitled to reception on any other grounds than those of an abstract *à priori* character, such as those which we have already indicated. *If* it be admitted as possible ("there is great virtue in an *if*,") that *any* combination of inorganic matter can, under *any* circumstances, produce a living being, we do not see why we should not look to such a combination as the real origin of *every* race, including man himself; each species thus being called into existence by the original ordination of the Creator, just when that coincidence of circumstances occurred which was favorable to its development and continuance. We see nothing more difficult in the supposition, that the *highest* animals had such an origin, than in attributing the same to the lowest; it is the origin of *a living cell* that constitutes the great stumbling-block; *ce n'est que le premier pas qui coute.* "Give me a vesicle," quoth Raspail, in the Archimedian style, "and I will make a thinking man." In this manner we account for the fact, that organic life seems to have *pressed in,* as our author has well phrased it, whenever the changing conditions of the surface of the globe rendered it capable of supporting new races, whilst they extinguished the old ones; without any twisting of the facts into a doctrine of progressive development. In this manner, too, we would account for the successive appearance of new species of infusoria, of a higher and higher type, in a vegetable infusion; the new ones making their appearance, as the character of the infusion became adapted to generate them, by new combinations of elements, rather than by the metamorphosis of forms which had been previously developed in accordance with a different state of things. And this view, moreover, seems to us much more consonant with the fact—*if* fact it be, that the being produced in Mr. Crosse's electric apparatus was not a monad, nor any other protozoic infusory, but an animal of complex organization, and capable of propagating its own kind. That the Creator formed man out of the dust of the earth, we have scriptural authority for believing; and we must confess our own predilection for the idea that, at a period however remotely antecedent, the Creator endowed certain forms of inorganic matter with the properties requisite to enable them to combine, at the fitting season, into the human organism,—over that which would lead us to regard the great-grandfather of our common progenitor as a chimpanzee or an oran-outang. We may here notice, by the way, another error of detail into which our author has fallen, in a later part of his work; where, after endeavouring to trace the origin of the human race to India, he remarks that, according to his theory of development, "we should expect man to have originated where the highest species of quadrumana are to be found;—these are unquestionably found in the Indian Archipelago." (p. 296.) Now, it has been clearly proved, by the elaborate comparison instituted by Proffessor Owen, that the oran-outang does not present nearly so striking an approach to the human race, as that which is exhibited by the chimpanzee, an inhabitant of *Western Africa;* and, further, that, in what are commonly regarded as the *lowest* varieties of the human race, the resemblance to the apes is in many points *less* than in the highest; as, for example, in the case of the development of the os calcis, which every one knows to be much greater in the Negro than in the European, whilst there is a complete absence of *heel* in the apes. So also, whilst the prominent muzzle of the Negro races must be regarded as a character of degradation, assimilating them to the lower tribes of animals, the large development of the cranium *behind* the foramen magnum, in the Negro, is anything but a point of affinity to the chimpanzee, oran-outang, or any other quadrumanous animal, since the foramen magnum is placed farther back, in the base of *their* crania, than it is in those of any of the human races.

Our limits forbid our dwelling any longer on these topics, though we are strongly attracted to do so by their highly interesting nature; and having devoted so much space to the discussion of our author's general views, we feel ourselves under the necessity of passing over, at least for the present, the remainder of his work, in which the application of these views to the physical and moral condition of man is displayed. We cannot conclude, however, without remark that the introduction of a chapter on the Macleay, or Quinary, system of animated nature, strikes us as the greatest blemish in the book. Although doubtless containing some germs of truth, and possessing the merit of having directed the attention of naturalists to more philosophical views of arrangement than those which formerly prevailed, we consider ourselves warranted in asserting,—as well from no small amount of consideration on our own parts, as from what we know to be the opinion of some of the most eminent naturalists of our own and other countries,—that, as a *natural system*, it is a most egregious failure, and that nothing but a most procrustean application of it can bring all the divisions and subdivisions of the animal kingdom to the number *five*. It bears upon the face of it so many marks of being a system of *human* invention, that we cannot for a moment imagine it to be the plan, on which the Creator has worked in the production of the several combinations which are presented to us in the animal kingdom. That *such a plan does exist*, every philosophic naturalist believes; and that it will be ultimately discovered, or that a considerable approach to it will be made, we have every reason to hope. But its discovery will be most assuredly retarded by hasty generalization; and our present amount of knowledge of a great proportion of the species composing the animal kingdom is yet too small to enable us to speculate on the subject with any considerable degree of probability,—far less, to entitle us to assume the truth of *any* of the numerous systems which have been proposed, as a basis for further argument. It would have answered our author's purpose nearly, if not to the full, as well, if he had assumed that *there is a regular system* in animated nature; which we believe that no one who has given the least attention to the subject would be hardy enough to contradict.

